# Tumor Suppressive Role of MUC6 in Wilms Tumor *via* Autophagy-Dependent β-Catenin Degradation

**DOI:** 10.3389/fonc.2022.756117

**Published:** 2022-04-28

**Authors:** Bai-Hui Liu, Gong-Bao Liu, Bin-Bin Zhang, Jian Shen, Lu-Lu Xie, Xiang-Qi Liu, Wei Yao, Rui Dong, Yun-Li Bi, Kui-Ran Dong

**Affiliations:** Department of Pediatric Surgery, Shanghai Key Laboratory of Birth Defect, Children’s Hospital of Fudan University, Shanghai, China

**Keywords:** Wilms tumor, MUC6, autophagy, β-catenin, whole genome sequencing

## Abstract

Wilms tumor is the most common renal malignancy in children. Known gene mutations account for about 40% of all wilms tumor cases, but the full map of genetic mutations in wilms tumor is far from clear. Whole genome sequencing and RNA sequencing were performed in 5 pairs of wilms tumor tissues and adjacent normal tissues to figure out important genetic mutations. Gene knock-down, CRISPR-induced mutations were used to investigate their potential effects in cell lines and *in-vivo* xenografted model. Mutations in seven novel genes (MUC6, GOLGA6L2, GPRIN2, MDN1, MUC4, OR4L1 and PDE4DIP) occurred in more than one patient. The most prevalent mutation was found in MUC6, which had 7 somatic exonic variants in 4 patients. In addition, TaqMan assay and immunoblot confirmed that MUC6 expression was reduced in WT tissues when compared with control tissues. Moreover, the results of MUC6 knock-down assay and CRISPR-induced MUC6 mutations showed that MUC6 inhibited tumor aggression *via* autophagy-dependent β-catenin degradation while its mutations attenuated tumor-suppressive effects of MUC6. Seven novel mutated genes (MUC6, GOLGA6L2, GPRIN2, MDN1, MUC4, OR4L1 and PDE4DIP) were found in WT, among which MUC6 was the most prevalent one. MUC6 acted as a tumor suppressive gene through autophagy dependent β-catenin pathway.

## Introduction

Wilms tumor (WT), also known as nephroblastoma, is the most common renal malignancy in children and it accounts for 5% of all childhood cancers (1). Its prevalence is around one in 10,000 in children under the age of 15. The standardized treatment for WT includes combination of surgery and chemotherapy, with the addition of radiotherapy in high-risk patients (2). The prognosis is generally good as its overall survival rate is 90%. However, the outcomes remain poor for the children with advanced tumors and it urges comprehensive investigation into the molecular mechanism underlying WT pathogenesis.

So far, huge efforts have been made to reveal the genetic landscape of WT. It’s well known that a few genes like WT1, CTNNB1 and WTX are somatically mutated in WT, but they account for about 30% of all WT cases (3). In other words, near 70% of WT cases have no known somatic mutation and the discovery of additional gene mutations will bring new insight into WT pathogenesis and uncover potential therapeutic targets. Indeed, recent sequencing studies show that mutations in DROSHA, DICER1, DGCR8, SIX1/2, MLLT1 are associated with WT (4–6). The US National Cancer Institute’s Therapeutically Applicable Research to Generate Effective Treatments (TARGET) initiative has revealed even more novel mutations in WT (7). It highlights the heterogeneity and complexity of WT genetics and the full map of genetic mutations in WT is far from clear.

In this study, we identified novel somatic mutations of MUC6 in WT tumors by whole genome sequencing. MUC6 mutations occur in 80% of WT samples (4/5) and a total of 7 nonsynonymous mutations were distributed in the C-terminal domain of MUC6 protein. In addition, we used MUC6 knock-down and CRISPR-induced MUC6 mutations to investigate the potential effects of MUC6 in cell lines. The results show that MUC6 inhibited tumor aggression *via* autophagy- dependent β-catenin degradation and MUC6 mutations compromised autophagy- dependent β-catenin degradation as well as the tumor-suppressive effects of MUC6. Thus, our study provides evidence that MUC6 play important roles in WT pathogenesis.

## Materials and Methods

### Clinical Samples

All cases were confirmed by pathological diagnosis. WT tissues and adjacent normal tissue were collected freshly during surgery, immediately stored in liquid nitrogen for subsequent extraction of DNA, RNA and protein.

### Cell Cultures

Human embryonic kidney cell line HEK293 was maintained in Dulbecco’s Modified Essential Medium (DMEM) with 10% fetal bovine serum (FBS), 100 U/ml penicillin and 100 mg/ml streptomycin. WT cell line WiT49 were maintained in 1:1 high-glucose Dulbecco’s modified Eagle’s medium/nutrient mixture F-12, 10% fetal calf serum, 100 units/ml penicillin and 100 mg/ml streptomycin. Cells were cultured in a humidified atmosphere with 5% CO2 at 37°C.

### RNA Sequencing

RNA-seq libraries were prepared using the Illumina TruSeq RNA Sample Preparation Kit v.2 and sequenced on the Illumina HiSeq 2000 platform using 101-bp paired-end reads. RNA-seq reads were trimmed to remove low-quality bases and adaptor contamination using TrimGalore and mapped to the hs37d5 genome assembly with the GENCODE v.13 annotation as a transcript guide using Tophat. Expression values were reported as fragments per kilobase of exon per million fragments mapped (FPKM) as calculated by CuffDiff. Transcriptome data have been deposited in GEO (http://www.ncbi.nlm.nih.gov/geo) under accession numbers GSE138869. DESeq was used to identify the differential gene expression between groups. Hierarchical clustering was performed to show the distinguishable gene expression profiles among samples. Gene exhibiting fold change >2 and q<0.05 were selected as significantly differentially expressed mRNA.

### Whole Genome Sequencing

DNA library preparation for whole genome sequencing was carried out using Illumina, Inc. v2 protocols. In brief, 1-5 μg of genomic DNA was fragmented to ~300 bp insert-size with a Covaris device, followed by size selection through agarose gel excision. Alignment of reads to the National Center for Biotechnology Information Build 37 reference human genome assembly was performed by the CGI Cancer Sequencing service analytic pipeline Version 2. After alignment, Picard (http://broadinstitute.github.io/picard/, Version 4.1.0.0) was employed to mark duplicate reads and SAMtools (Version: 1.4) was used to convert alignment result format. GATK (Version 4.1.0.0) was used to call out all the variants, including SNPs and InDels. Then SnpEff was applied to annotate all the variants. Sequencing data are accessible in SRA (https://www.ncbi.nlm.nih.gov/sra) under accession number SRP225389.

### Gene Function Analysis

The predicted target genes were put into the Database for Annotation, Visualization and Integrated Discovery (http://david.abcc.ncifcrf.gov/) for the annotation and molecular function by using the Gene Ontology (GO). The Kyoto Encyclopedia of Genes and Genomes (KEGG) database (http://www.genome.ad.jp/kegg/) was used to identify the potential functions of these target genes in the pathways. P<0.05 was used as the cut−off value.

### Immunoblot

Cells were lysed in radioimmunoprecipitation assay (RIPA) buffer and the protein concentration was measured using the bicinchoninic acid (BCA) protein assay (Pierce, Rockford, IL, USA). Proteins were separated by sodium dodecyl sulfate polyacrylamide gel electrophoresis (SDS-PAGE) and transferred to Hybond ECL nitrocellulose membranes (Amersham Biosciences, Little Chalfont, Buckinghamshire, UK). Following primary antibodies were used: MUC6 (ab192318, abcam, 1:500 dilution), β-catenin (8480, CST, 1:1000 dilution), c-Myc (5605, CST, 1:1000 dilution), Survivin (2808, CST, 1:1000 dilution), MMP-7 (3801, CST, 1:1000 dilution), Atg5 (12994, CST, 1:1000 dilution), Atg7 (8558, CST, 1:1000 dilution), Beclin-1(3495, CST, 1:1000 dilution), LC3A/B (12741, CST, 1:1000 dilution). The membranes were then incubated with near-infrared fluorescent secondary antibodies for 2h. Protein bands were visualized using the LI-COR Odyssey System (LI-COR Biotechnology, USA).

### Quantitative Real-Time PCR

Quantitative real time-PCR (qPCR) was performed by TaqMan assay to measure the relative mRNA levels of MUC6 (#4331182) and β-catenin (#4331182). Briefly, Total RNA was extracted from tissues or cell lines using TRIzol reagent and the cDNA was generated with 1 ug total RNA, reverse transcriptase and random primers. Actin was used as an endogenous control. The relative fold change for each target gene compared to the control group was calculated using the ΔΔCt method.

### Over-Expression and Knock-Down of MUC6

For over-expression experiments, the coding sequences of N-terminal (1-800 aa) and C-terminal (1200-2000 aa) of human MUC6 were amplified by PCR, fused with signal peptide at N-terminal and HA tag at C-terminal, and cloned into pLenti-EGFP vector, respectively. To perform MUC6 knock-down, two independent the short hairpin RNAs (shRNAs) targeting human MUC6 (NM_005961.3) sequence were designed. Their sequences were as follows. MUC6 shRNA-1, forward: TGGAGGTCACAATAGCTGCCTGCCAAATTCAAGAGA TTTGGCAGGCAGCTATTGTGACCTCCTTTTTTC; reverse: TCGAGAAAAAAGGAGGTCA CAATAGCTGCCTGCCAAATCTCTTGAATTTGGCAGGCAGCTATTGTGACCTCCA. MUC6 shRNA-2, forward: TGCACAACTAATCAGCTGTCCTCCTCATTCAAGAGATGAG GAGGACAGCTGATTAGTTGTGCTTTTTTC; reverse: TCGAGAAAAAAGCACAACTAATC AGCTGTCCTCCTCATCTCTTGAATGAGGAGGACAGCTGATTAGTTGTGCA. Target sequences were underlined. The scramble sequence was used as control. They were constructed into the pLentiLox3.7 (pLL3.7) lentiviral vector, respectively. The lentivirus was packaged and amplified in HEK293T cells. Cell lines were infected at an MOI of 5.

### CRISPR-Mediated Mutation of MUC6

CRISPR-mediated editing in WiT49 cell was performed with pLenti-Cas9-P2A-tGFP from Origene and gRNAs targeting indicated sites were cloned into this vector. WiT49 cells were infected by pLenti-Cas9-P2A-tGFP with targeting gRNA or scramble control in the presence of donor DNA for 72 hours. Flow cytometry was used to sort single GFP-positive cell into 96-well plate and individual clones were expanded. Genomic DNA was isolated from individual clones and gene editing was confirmed by Sanger DNA sequencing.

### *In-Vivo* Xenografted Model

The animal experiments were conducted in accord with the institutional animal welfare guidelines. Female athymic nude mice (6 weeks old, BALB-c/nu/nu strain) were kept in specific pathogen-free condition and were randomly assigned to three groups: control WiT49 cell (n=5), MUC6 mutant WiT49 cell (n=5) and MUC6 mutant WiT49 cell plus DN-β-catenin (n=5), 5 animals per group. A total of 1×10^6^ cells were implanted into subcutaneous tissue of posterior limb with a 26-gauge needle/1 ml syringe. Tumor size was measured after 4 weeks by measuring the length (L) and width (W) of xenografted tumors with a vernier caliper. Tumor size was calculated as follows: L×(W)^2/^2.

### Cell Proliferation Assay

Cells were seeded into a 96-well plate in triplicate at the concentration of 4×10^3^ cells per well. The cell growth was measured by 3-(4,5-dimethylthiazol-2-yl)- 2,5-diphenyltetrazolium (MTT) bromide assay at day 1, 2, 3, 4 and 5, respectively. Cells were incubated with 5 mg/ml MTT for 4 h, and subsequently solubilized in DMSO (100 ul/well). The absorbance at 570 nm was then measured using an ELISA reader.

### Transwell Invasion Assay

Cell invasion assay was performed in transwell chamber. Cells (1×10^5^) were suspended in 200 uL serum-free medium and seeded into the upper chamber of a transwell inserted with an 8-μm pore size membrane and coated with matrigel (Sigma). Culture medium containing 20% FBS was placed in the lower chamber. After incubating for 48h, the non-invading cells were removed with cotton swabs. Invasive cells at the bottom of the membrane were stained with 0.5% crystal violet and were counted under a microscope. The data were presented as fold change relative to the control group.

### Apoptosis

Cells were trypsinized, washed, and stained with Annexin V-PE Apoptosis kit (abcam, ab14155) in the dark for 15 min at room temperature. Then, the stained cells were analyzed by MoFlo XDP (Beckman Coulter, Inc).

### Luciferase

For MUC6 promoter luciferase assay, 2500bp sequence of human MUC6 promoters was amplified by PCR and cloned into pGL4.10 vector. For MUC6 3’UTR luciferase assay, full-length sequence of human MUC6 3’UTR (632 bp) was amplified by PCR and cloned into pMIR-report. Renilla luciferase vector was used to normalize for transfection efficiency. For luciferase reporter assay, cells were transiently transfected with luciferase vectors using Lipofectamine 2000 for 48h. Reporter activity was measured by the dual-luciferase assay-system (Promega). The data were presented as fold change relative to the control group.

### Statistics

Statistical analysis was performed using GraphPad Prism software. All data were presented as mean ± SD and statistical analysis was performed by two-tailed Student t test for two groups and one way ANOVA with Newman-Keuls *post hoc* test for more than two groups. Statistically significant differences were defined as P < 0.05. For all, *P<0.05, **P<0.01, ***P<0.001.

## Results

### Whole Genome Sequencing of WT Samples

**Figure 1:**
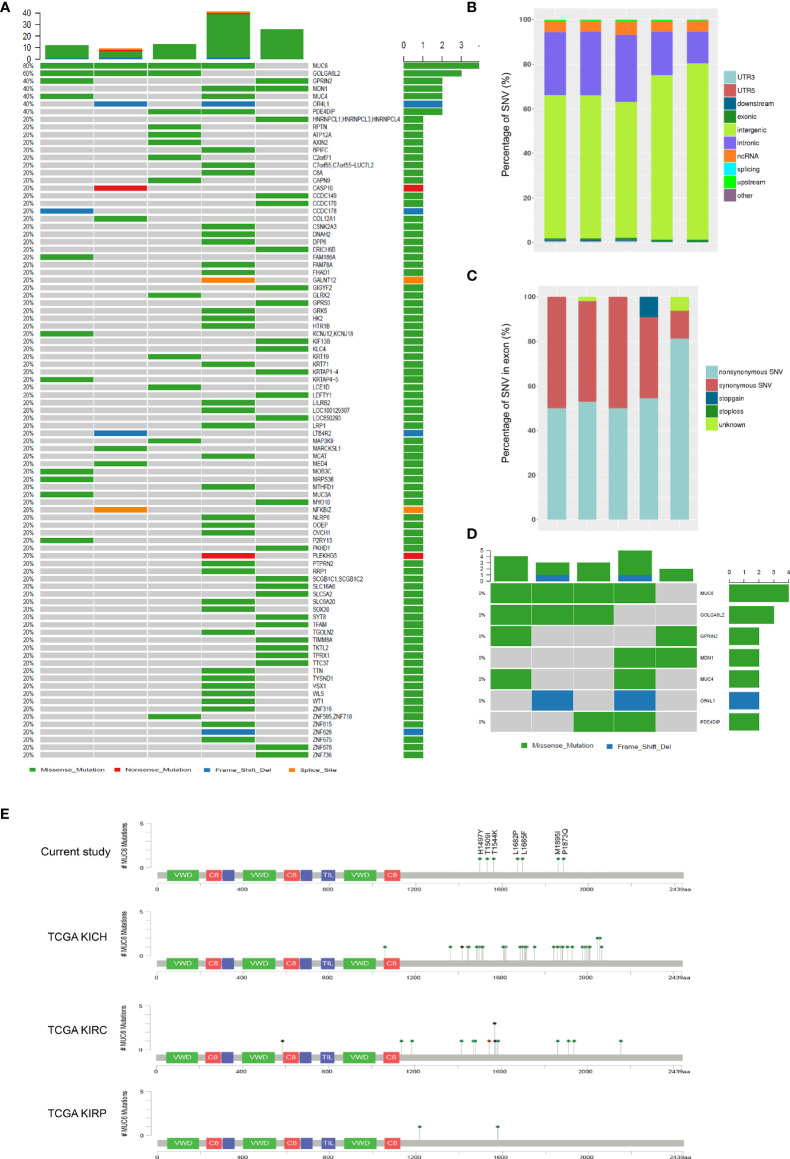
Whole genome sequencing of WT samples. **(A)** SNVs in 5 fresh-frozen WT samples. The colored squares refer to the corresponding types of SNVs (missense, nonsense, frame shift indel and splice site). **(B)** The genomic distribution of all SNVs. **(C)** The functional category of all SNVs in exon. **(D)** SNVs in 7 genes which were mutated twice or more. **(E)** Schematic representation of human MUC6 protein, showing the positions of 7 identified mutations in current study and MUC6 mutations in TCGA KICH, KIRC and KIRP datasets.

Whole genome sequencing was performed in 5 WT tumors and their adjacent control tissues. **Table 1** shows the patient demographics information. Bioinformatics analysis identified 95 high-quality somatic, nonsynonymous small exonic variants, with an average of 19 candidate mutations per case (range from 7 to 36 mutations per case). This low rate of somatic alterations was expected as embryonic tumors tend to have fewer somatic mutations. A total of 91 genes were found to be mutated at least once (**Figure 1A**) and the genomic distribution and functional category of all single nucleotide variants (SNV) were shown in **Figures 1B, C**, respectively. A detailed description of SNVs in each patient was provided in **Additional Files 1**–**5**. However, only 7 genes were mutated twice or more (**Figure 1D**) and they were MUC6 (n=4), GOLGA6L2 (n=3), GPRIN2 (n=2), MDN1 (n=2), MUC4 (n=2), OR4L1 (n=2) and PDE4DIP (n=2). None of them were reported in WT by previous studies.

**Table 1:** Demographics of WT cases for whole genome sequencing and RNA sequencing.

Number	Gender	Age	Pathology
P1	Male	6Y11M	Favorable Histology
P2	Female	1Y4M	Favorable Histology
P3	Female	1Y6M	Favorable Histology
P4	Male	4Y5M	Favorable Histology
P5	Female	1Y5M	Favorable Histology

Consistent with previous studies, somatic mutations were found in WT1 (1 patient) and CTNNB1 (1 patients). Unexpectedly, the most prevalent somatic mutation was found in MUC6. A total of 7 somatic exonic variants were identified in MUC6 gene in 4 patients (80%). One tumor had MUC6 mutation c.C4489T, encoding p.H1497Y. One tumor had MUC6 mutation c.T5045C, encoding p.L1682P. One tumor had two MUC6 mutations: c.A5055C, encoding p.L1685F and c.C4631A, encoding p.T1544K. One tumor had three MUC6 mutations: c.G5685C, encoding p.M1895I, c.C5618A, encoding p.P1873Q and c.C4526T, encoding p.T1509I. As shown in **Figure 1E**, the identified MUC6 mutations were enriched in the C-terminal domain of MUC6 gene. Then, we explored The Cancer Genome Atlas (TCGA) database and found that MUC6 mutations also occur in TCGA KICH (Kidney Chromophobe), KIRC (Kidney renal clear cell carcinoma) and KIRP (Kidney renal papillary cell carcinoma) datasets (**Figure 1E**). Interestingly, the MUC6 mutations in these TCGA datasets were also mainly located in the C-terminal domain of MUC6.

**Figure 2:**
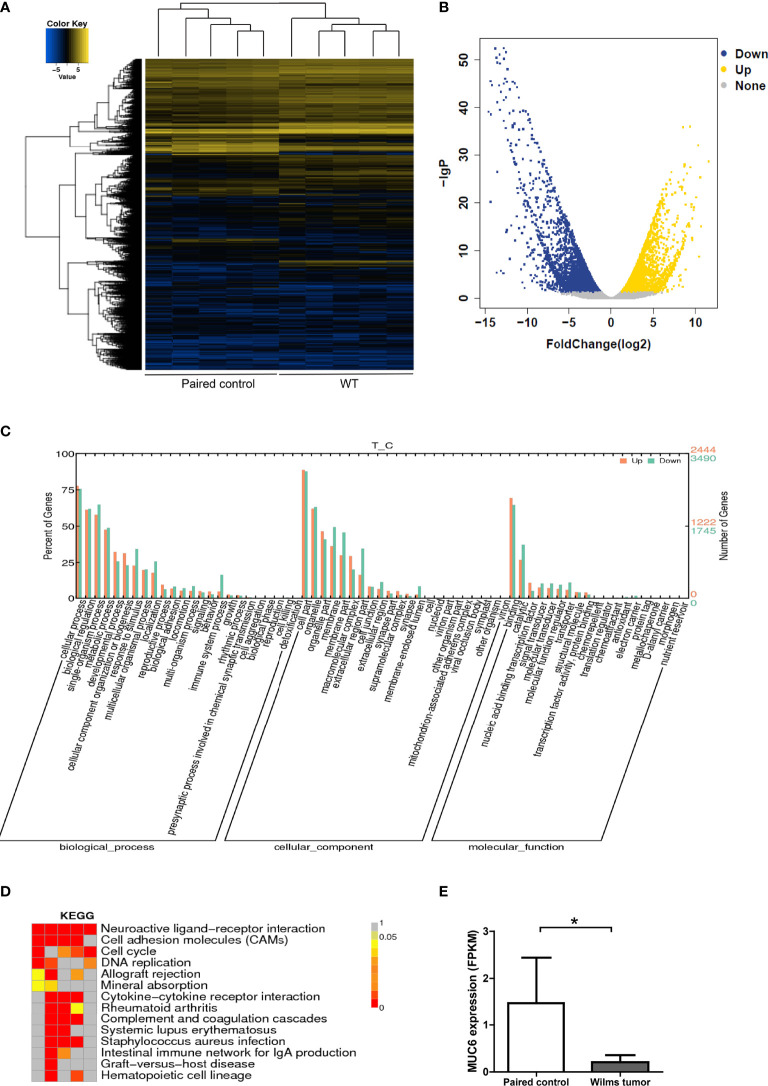
RNA sequencing of WT samples. **(A)** Heatmap showing the gene expression profiling of 5 pairs of WT samples. **(B)** Volcano plot of the differential gene expression. **(C)** Gene Ontology annotation showing the biological process, cellular component and molecular function of differentially expressed genes. **(D)** Significantly enriched KEGG pathways. **(E)** MUC6 mRNA level revealed by RNA sequencing in WT tissues and paired control tissues. *P<0.05.

In the meantime, global gene expression was explored by RNA sequencing in the same 5 pairs of WT and control tissues (**Figure 2A**). Volcano plot of the differential gene expression was shown in **Figure 2B**. Gene Ontology annotation showed the biological process, cellular component and molecular function of differentially expressed genes (**Figure 2C**). Significantly enriched KEGG (Kyoto Encyclopedia of Genes and Genomes) pathways were shown in **Figure 2D**. The FPKM expression data of 5 pairs of WT tissues were provided in **Supplementary File 6**. It’s important to note that MUC6 mRNA level was also reduced in WT tissues compared to control tissues (**Figure 2E**).

### MUC6 Expression and Its Correlation With β-Catenin in WT Tumor

**Figure 3:**
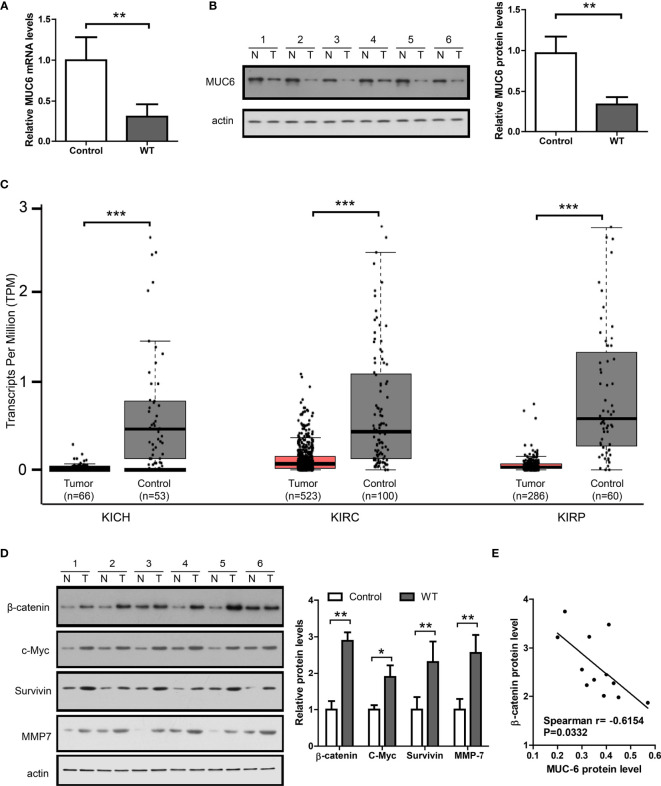
MUC6 expression and its correlation with β-catenin signaling in WT tumor. **(A)** Real-time PCR results showing MUC6 mRNA levels in 12 pairs of WT tissues. **(B)** Immunoblots showing MUC6 protein levels in 12 pairs of WT tissues. N indicates normal control tissues and T indicates tumor tissues. **(C)** whiskers-box plots showing MUC6 expression levels in indicated TCGA datasets. **(D)** Immunoblots showing protein levels of β-catenin and its downstream target genes including c-Myc, Survivin and MMP7 in 12 pairs of WT tissues. **(E)** Scatter plot showing the association of MUC6 protein level with β-catenin in WT tumor tissue (Spearman r=-0.6154, P=0.0332). For all, *P<0.05; **P<0.01; ***P<0.001.

To measure MUC6 expression, qPCR and immunoblot were performed in 12 WT tumors and matched control tissues. The results of TaqMan assay showed that MUC6 mRNA levels were reduced in WT tissues compared to matched control tissues (**Figure 3A**). The representative image of immunoblot and its quantification (**Figure 3B**) show that MUC6 protein levels were also reduced in WT tissues compared to matched control tissues. We also analyzed TCGA pan-kidney cancer datasets including KICH, KIRC and KIRP. The expression data from these sets showed a robust reduction of MUC6 in kidney tumor tissues compared to control tissues (**Figure 3C**).

To explore the potential association of MUC6 with β-catenin signaling pathway, the protein levels of β-catenin and its downstream target genes including c-Myc (8), MMP7 (9) and survivin (10) were measured by immunoblot in the same 12 pairs of samples. The results show that β-catenin, c-Myc, MMP7 and survivin levels were significantly higher in WT tissues compared to control tissues (**Figure 3D**) and there was a significant correlation between MUC6 protein level and β-catenin protein level in WT tissues (**Figure 3E**). Taken together, these results suggest that MUC6 expression was down-regulated in WT tissues and MUC6 level was negatively correlated with β-catenin in WT tissues. Thus, MUC6 might regulate β-catenin level in WT.

### MUC6 Induces β-Catenin Degradation *via* Autophagy Pathway

**Figure 4:**
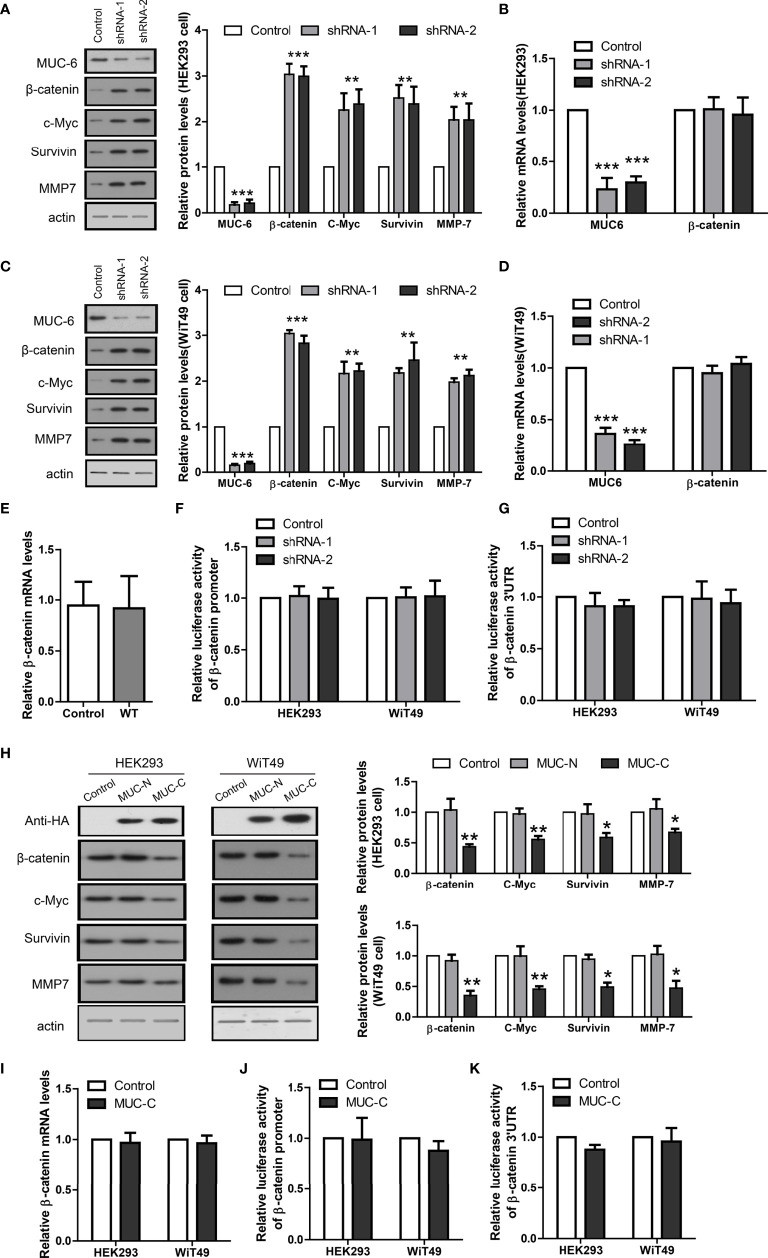
MUC6 reduces β-catenin protein level. **(A)** Immunoblots showing protein levels of MUC6, β-catenin, c-Myc, Survivin and MMP7 in HEK293 cell infected with indicated shRNAs. **(B)** Real-time PCR results showing the mRNA levels of MUC6 and β-catenin in HEK293 cell infected with indicated shRNAs. **(C)** Immunoblots showing protein levels of MUC6, β-catenin, c-Myc, Survivin and MMP7 in WiT49 cell infected with indicated shRNAs. **(D)** Real-time PCR results showing the mRNA levels of MUC6 and β-catenin in WiT49 cell infected with indicated shRNAs. **(E)** Real-time PCR results showing β-catenin mRNA levels in 12 pairs of WT tissues. **(F)** Luciferase assays showing the activity of β-catenin promoter in cells infected with indicated shRNAs. **(G)** Luciferase assays showing the activity of β-catenin 3’UTR in cells infected with indicated shRNAs. **(H)** Immunoblots showing protein levels of β-catenin, c-Myc, Survivin and MMP7 after over-expression of MUC6-N or C terminal in HEK293 and WiT49 cell lines. **(I)** Real-time PCR results showing β-catenin mRNA levels after MUC6-C over-expression in HEK293 and WiT49 cell lines. **(J)** Luciferase assays showing the activity of β-catenin promoter in HEK293 and WiT49 cell lines after MUC6-C over-expression. **(K)** Luciferase assays showing the activity of β-catenin 3’UTR in HEK293 and WiT49 cell lines after MUC6-C over-expression. For all, *P<0.05; **P<0.01; ***P<0.001.

To provide direct evidence that MUC6 regulates β-catenin level in WT, we knock-down endogenous MUC6 in human kidney cell line HEK293 and human WT cell line WiT49, respectively. To avoid potential off-target effect, we designed two independent shRNAs against human MUC6 and evaluated their efficiency in HEK293 and WiT49 cells, respectively. The immunoblot results showed that MUC6 shRNAs greatly down-regulated MUC6 protein levels (**Figures 4A, C**). In addition, MUC6 knock-down increased the protein levels of β-catenin and its target genes c-Myc, MMP7 and survivin in both cell lines. However, qRT-PCR results showed that MUC6 knock-down had no effect on β-catenin mRNA level (**Figures 4B, D**). This is consistent with the result that there was no difference in β-catenin mRNA level in our 12 pairs of WT and control tissues (**Figure 4E**). In addition, MUC6 knock-down had no effect on the β-catenin promoter activity in luciferase assay (**Figure 4F**). It suggests that MUC6 knock-down didn’t affect β-catenin transcription. Similarly, MUC6 knock-down showed no effect on the β-catenin 3’UTR activity in luciferase assay (**Figure 4G**). It suggests that MUC6 is unlikely to regulate β-catenin expression through microRNAs which target its 3’UTR.

As the very large size of MUC6 makes its full-length over-expression difficult and previous study showed that C terminal of MUC6 alone is sufficient to impose biological effects (11), we cloned its N-terminal (MUC-N, 1-800aa) and C-terminal (MUC-C, 1200-2000aa) fragments, fused them with N-terminal signal peptide and C-terminal HA tag, and over-expressed them in HEK293 and WiT49 cells, respectively. MUC-C, but not MUC-N, reduced the protein levels of β-catenin and its target genes c-Myc, MMP7 and survivin (**Figure 4H**). Consistently, MUC-C over-expression had no effect on β-catenin mRNA level (**Figure 4I**), promoter activity (**Figure 4J**) or 3’UTR activity (**Figure 4K**). Taken together, the results from MUC6 knock-down and MUC-C over-expression suggest that MUC6 suppressed β-catenin protein level likely *via* post-translational mechanism.

**Figure 5:**
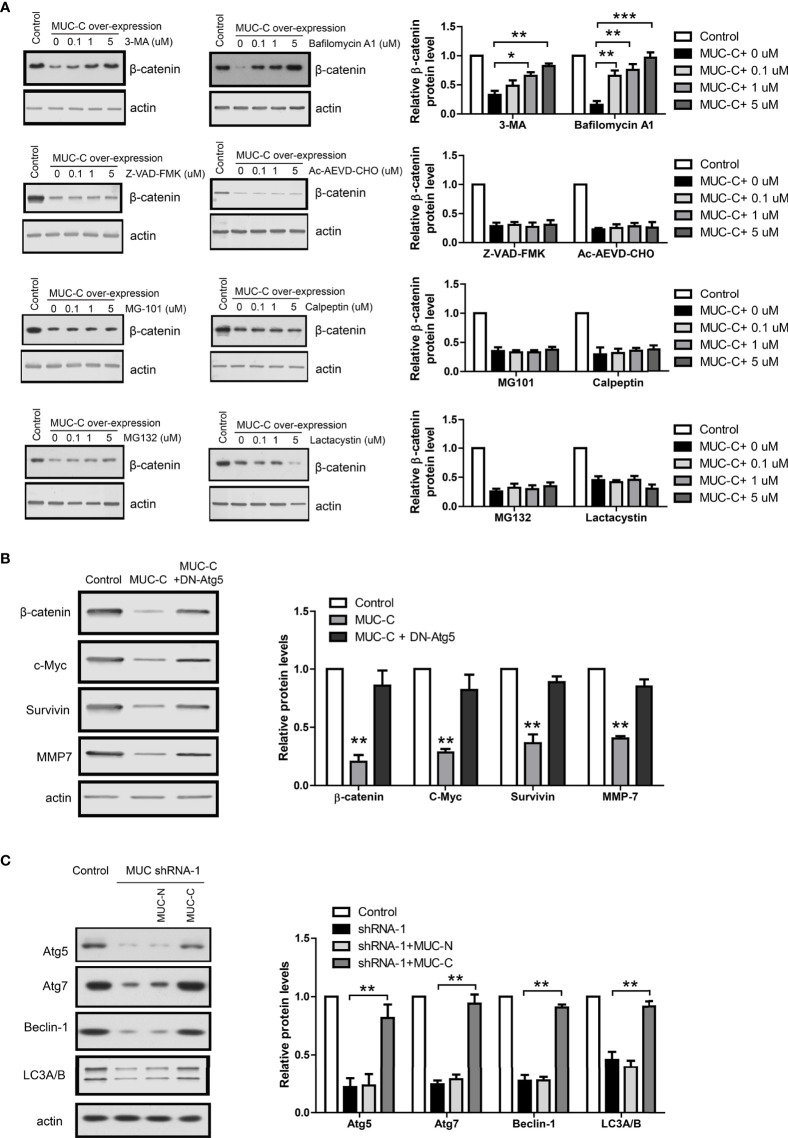
Autophagy pathway mediates MUC6-induced β-catenin degradation in WT cell. **(A)** Immunoblots showing β-catenin protein levels in WiT49 cell treated with Z-VAD-FMK, Ac-AEVD-CHO, MG-101, Calpeptin, MG132, Lactacystin, 3-MA or Bafilomycin A1 at indicated concentrations. **(B)** Immunoblots showing β-catenin protein levels in WiT49 cell infected with MUC-C alone or with dominant negative Atg5 together. **(C)** Immunoblots showing the effects of MUC-N or MUC-C over-expression on Atg5, Atg7, Beclin-1 and LC3A/B protein levels in WiT49 cell infected with MUC shRNA-1. For all, *P<0.05; **P<0.01; ***P<0.001.

Thus, we treated MUC-C over-expressing WiT49 cell with following proteinase inhibitors under a wide concentration range (0, 0.1, 1, 5 u M): Z-VAD-FMK and Ac-AEVD-CHO, the caspase inhibitors; MG-101 and Calpeptin, the calpain inhibitors; MG132 and Lactacystin, the proteasome inhibitors; 3-MA and Bafilomycin A1, the autophagy inhibitors. The immunoblot results show that only autophagy inhibitors could fully rescue MUC-C induced β-catenin degradation in a dose-dependent way (**Figure 5A**). To further confirm the above results from chemical inhibitors, we used a dominant negative Atg5 mutant (DN-Atg5) to inhibit autophagy (12). Consistently, DN-Atg5 also rescued MUC-C induced β-catenin degradation and β-catenin target genes (**Figure 5B**). In addition, MUC6 knock-down led to down-regulation of autophagy markers like Atg5, Atg7, Beclin-1 and LC3A/B which could be rescued by the over-expression of MUC6-C but not MUC-N (**Figure 5C**). Taken together, the above results support that MUC6 induces β-catenin degradation *via* autophagy pathway.

### MUC6 Mutations Compromise Autophagy-Dependent β-Catenin Degradation

**Figure 6:**
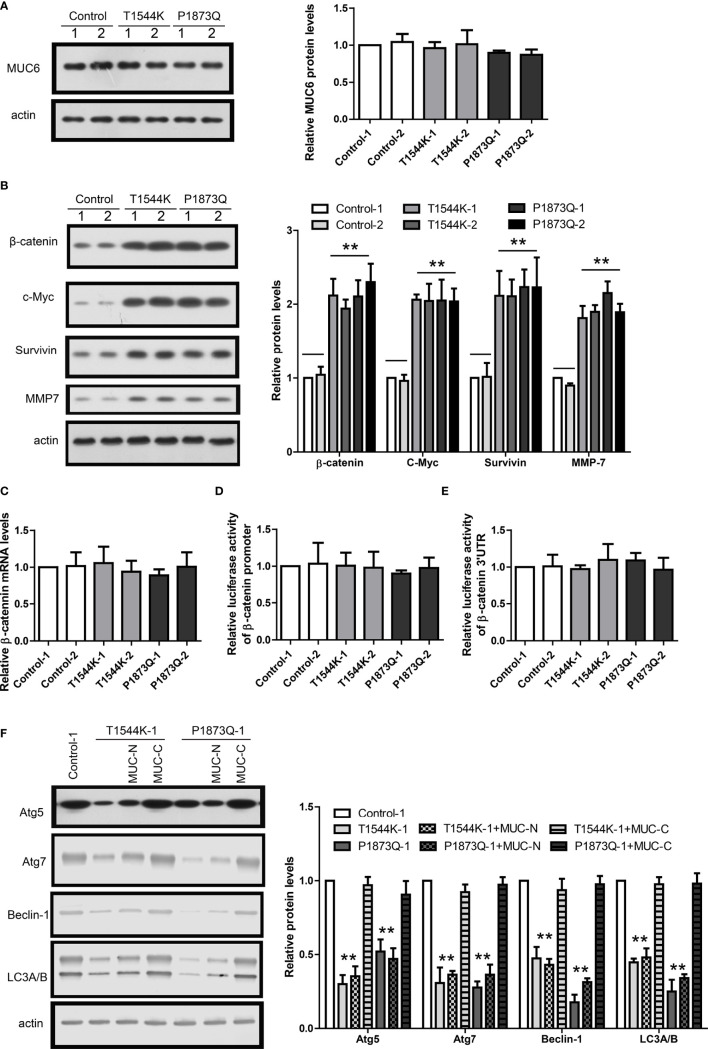
MUC6 mutations compromise autophagy-dependent β-catenin degradation. **(A)** Immunoblots showing MUC6 protein levels in two clones from isogenic control, T1544K mutant and P1873Q mutant WiT49 cells, respectively. **(B)** Immunoblots showing β-catenin, c-Myc, Survivin and MMP7 protein levels in two clones from isogenic control, T1544K mutant and P1873Q mutant WiT49 cells, respectively. **(C)** Real-time PCR results showing β-catenin mRNA levels in two clones from isogenic control, T1544K mutant and P1873Q mutant WiT49 cells, respectively. **(D)** Luciferase assays showing the activity of β-catenin promoter in two clones from isogenic control, T1544K mutant and P1873Q mutant WiT49 cells, respectively. **(E)** Luciferase assays showing the activity of β-catenin 3’UTR in two clones from isogenic control, T1544K mutant and P1873Q mutant WiT49 cells, respectively. **(F)** Immunoblots showing the effects of MUC-N or MUC-C over-expression on Atg5, Atg7, Beclin-1 and LC3A/B protein levels in one clone from isogenic control, T1544K mutant and P1873Q mutant WiT49 cells, respectively. For all, **P<0.01.

To recapitulate the identified MUC6 mutations, we used CRISPR to induce T1544K and P1873Q mutations into WiT49 cell, respectively. To avoid potential difference among individual cell clones, two clones of each MUC6 mutant cell and two clones of isogenic control cell were used. The homozygous mutations were confirmed by sanger DNA sequencing. The expression of MUC6 mutant protein was confirmed by immunoblot and the results show that MUC6 protein levels in MUC6 mutant cells were comparable to those in isogenic control cell (**Figure 6A**).

In MUC6 mutant cells, the protein levels of β-catenin and its target genes were increased compared to control cell (**Figure 6B**). In contrast, β-catenin mRNA level was not different between MUC6 mutant cells and control cell (**Figure 6C**). In addition, luciferase assay showed no difference on the β-catenin promoter activity (**Figure 6D**) or 3’UTR activity (**Figure 6E**) between MUC6 mutant cells and control cell. Similar to MUC6 knock-down, MUC6 mutations also led to down-regulation of Atg5, Atg7, Beclin-1 and LC3A/B (**Figure 6F**). Finally, the down-regulation of autophagy markers in MUC6 mutant cells could be rescued by the over-expression of MUC6-C but not MUC-N (**Figure 6F**).

Taken together, the results in MUC6 mutant cells were consistent to those of MUC6 knock-down and they suggest that the MUC6 mutations identified in our WT tissues could compromise the autophagy-dependent β-catenin degradation.

### MUC6-Autophagy-β-Catenin Regulates Tumor Behaviors in *In-Vitro* Assays

**Figure 7:**
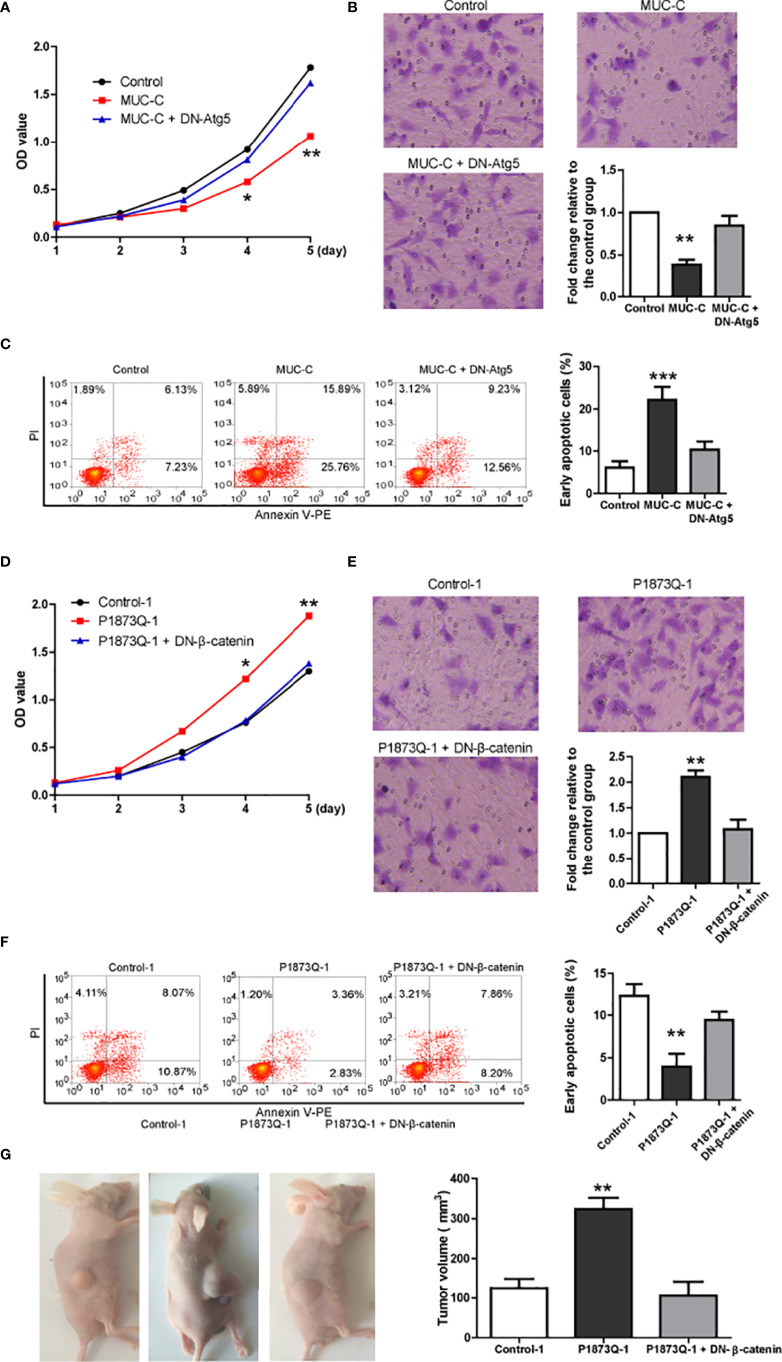
MUC6-autophagy-β-catenin regulates tumor behaviors in *in-vitro* assays. **(A)** MTT assay showing the proliferation of WiT49 cell infected with indicated vectors. **(B)** Transwell invasion assay showing the invasion of WiT49 cell infected with indicated vectors. **(C)** Annexin V apoptosis assay showing the apoptosis of WiT49 cell infected with indicated vectors. **(D)** MTT assay showing the proliferation of one clone from isogenic control cell and P1873Q mutant WiT49 cell, respectively. **(E)** Transwell invasion assay showing the invasion of one clone from isogenic control cell and P1873Q mutant WiT49 cell, respectively. **(F)** Annexin V apoptosis assay showing the apoptosis of one clone from isogenic control cell and P1873Q mutant WiT49 cell, respectively. For all, *P<0.05; **P<0.01; ***P<0.001. **(G)** Representative images of xenografted model and quantification of tumor size of three groups: isogenic control WiT49 cell (n=5), MUC6 P1873Q mutant WiT49 cell (n=5) and MUC6 P1873Q mutant WiT49 cell plus DN-β-catenin (n=5). **P<0.01.

As β-catenin is essential for WT tumorigenesis (13), MUC6 may affect tumor cell behaviors *via* autophagy-β-catenin pathway. Thus, we investigated the effects of MUC-C over-expression in WiT49 cell with various *in-vitro* assays: MTT proliferation assay, transwell invasion assay and Annexin V apoptosis assay. The results show that MUC-C over-expression could inhibit proliferation (**Figure 7A**), suppress cell invasion (**Figure 7B**) and increase the number of apoptotic cell (**Figure 7C**). As expected, all above effects were blocked by DN-Atg5.

Next, we compared the behaviors of MUC6 mutant (P1873Q) WiT49 cell to those of isogenic control WiT49 cell in the same assays. Consistently, mutant cell showed slower proliferation (**Figure 7D**), less cell invasion (**Figure 7E**) and more apoptotic cell (**Figure 7F**) compared to control cell. As expected, all above effects were rescued by DN-β-catenin (14).

### MUC6 Mutation Promotes *In-Vivo* Tumor Formation *via* β-Catenin

To demonstrate that MUC6 mutation have effects on *in-vivo* tumor formation, we used MUC6 mutant (P1873Q) WiT49 cell and control WiT49 cell to establish subcutaneously xenografted nude mice. Five nude mice were randomly assigned to each group: control WiT49 cell (n=5), MUC6 mutant WiT49 cell (n=5) and MUC6 mutant WiT49 cell plus DN-β-catenin (n=5). Four weeks after implantation, mice were sacrificed and the tumor tissues were collected. The results showed that tumors from MUC6 mutant WiT49 cell had much larger size compared to control cell, but DN-β-catenin dramatically decreased the tumor size of MUC6 mutant WiT49 cell (**Figure 7G**). Taken together, these results suggest that MUC6 mutation promotes *in-vivo* tumor formation *via* β-catenin.

## Discussion

Sequencing projects of various cancers have produced massive data and it provides unprecedented opportunity to explore the complex genetic landscapes of tumors including Wilms tumor (15). However, known mutations contribute to only about 40% of all WT cases. It suggests that novel mutations may be involved in majority WT cases. Here, we identified novel MUC6 mutations in WT through whole genome sequencing. RNA sequencing and further validation with TaqMan assay and immunoblot confirmed that MUC6 expression was reduced in WT tissues compared to control tissues. In addition, MUC6 expression was negatively correlated with β-catenin up-regulation in WT tissues. Next, in cultured cell lines, we used MUC6 know-down and CRISPR induced MUC6 mutations to demonstrate that MUC6 induced β-catenin degradation *via* autophagy pathway and MUC6 mutations compromised the autophagy-dependent β-catenin degradation. Finally, MUC6-autophagy-β-catenin pathway affects tumor behaviors in *in-vitro* and *in-vivo* assays. Taken together, in normal conditions, secreted MUC6 activates autophagy possibly *via* binding to unknown surface receptor. It leads to autophagy-dependent degradation of β-catenin. In this way, MUC6 plays a tumor-suppressive role in WT. However, when MUC6 expression is down-regulated or MUC6 mutations occur in WT, autophagy-dependent β-catenin degradation is impaired and β-catenin could activate its target genes to promote tumorigenesis. The tumor-suppressive effect of MUC6 in WT is generally consistent with its roles in gastric cancer (16, 17), colorectal cancer (18), intestinal ovarian mucinous neoplasms (19) and pancreatic cancer (11).

We noticed the high mutation rate of MUC6 in our WT samples. It may be simply a bias from our relative small sample size, but it’s important to note that the incidence rate of WT in Chinese population is much lower than that of western countries (20) and therefore Chinese WT patients may have unique genetic traits. MUC6 belongs to the Mucins family and it represents a group of large glycoproteins expressed mainly by epithelial cells and Mucins form a physical barrier to protect the epithelial cells and lubricate lumens of tubular organs (21). They are also involved in the differentiation and renewal of epithelial cells, cell adhesion, immune response and cell signaling (22). In fact, Mucins appear to be frequently mutated in various cancers (23). The novel mutations of MUC6 together with other genes GOLGA6L2, GPRIN2, MDN1, MUC4, OR4L1 and PDE4DIP identified in our study support that Chinese WT patients may have distinct underlying mechanism and it’s imperative to develop therapies against these novel targets.

The oncogenic role of β-catenin in WT is well established and a small portion of WT patients have mutations in its coding gene CTNNB1. However, most WT patients do not have CTNNB1 mutations and the regulation of β-catenin level in WT is poorly understood. Here, we first show that MUC6 reduced β-catenin protein level through post-translational mechanism. Then, we use a panel of pharmacological inhibitors to explore the proteinase responsible for β-catenin degradation. Surprisingly, we found that MUC6 induced autophagy-dependent degradation of β-catenin and β-catenin mediated the biological effects of MUC6 in various *in-vitro* and *in-vivo* assays. Not coincidently, previous studies have shown autophagy-mediated β-catenin degradation (24, 25). These independent studies are fully consistent with our current findings and support a novel regulation mechanism of MUC6-autophagy-β-catenin pathway in WT.

The major limitation in our study is that how MUC6 activates autophagy is unknown. Members of the human Mucin family, including MUC1 to MUC21, can be divided into two classes: secreted mucins (such as MUC-2 and MUC-6) and transmembrane mucins (such as MUC-1, MUC-4 and MUC-13). Transmembrane mucins have receptors like HER2 (26, 27) and ErbB2 (28, 29). As for secreted mucins, although no study reports that MUC6 has its own receptor so far, MUC2 interacts with galectin-3-Dectin-1-FcγRIIB receptor complex in small intestine (30). Thus, it’s possible MUC6 may function by binding to certain cell surface receptors. However, the large size and its abundant glyscans have made it difficult to produce and purify recombinant MUC6 and use it as bait to find out its potential receptors. It would be of great interest to look for those receptors in future studies.

Through whole-genome sequencing of 5 WT patients, we identified novel somatic *MUC6* mutations in WT, which inhibits β-catenin expression through autophagy-dependent degradation to further affect tumor behaviors. It reveals that *MUC6* plays an important tumor-suppressive role in WT, and thus may be a promising therapeutic target for WT.

## Data Availability Statement

The datasets presented in this study can be found in online repositories. The names of the repository/repositories and accession number(s) can be found below: NCBI [accession: GSE138869, SRP225389].

## Ethics Statement

Written informed consent to participate in this study was provided by the participants’ legal guardian/next of kin. Written informed consent was obtained from the minor(s)’ legal guardian/next of kin for the publication of any potentially identifiable images or data included in this article. The animal study was reviewed and approved by Ethics Committee of the Children’s Hospital of Fudan University.

## Author Contributions

K-RD and Y-LB contributed to the conception of the study. B-HL and G-BL performed the experiment and manuscript preparation. B-BZ and JS contributed significantly to analysis and manuscript preparation. L-LX and X-QL performed the data analyses and wrote the manuscript. WY and RD helped perform the analysis with constructive discussions. All authors contributed to the article and approved the submitted version.

## Funding

This work was received financial support from the Cyrus Tang Foundation, Shanghai Municipal Key Clinical Specialty (no. shslczdzk05703).

## Conflict of Interest

The authors declare that the research was conducted in the absence of any commercial or financial relationships that could be construed as a potential conflict of interest.

## Publisher’s Note

All claims expressed in this article are solely those of the authors and do not necessarily represent those of their affiliated organizations, or those of the publisher, the editors and the reviewers. Any product that may be evaluated in this article, or claim that may be made by its manufacturer, is not guaranteed or endorsed by the publisher.
